# Characteristics of Plasticized Lithium Ion Conducting Green Polymer Blend Electrolytes Based on CS: Dextran with High Energy Density and Specific Capacitance

**DOI:** 10.3390/polym13213613

**Published:** 2021-10-20

**Authors:** Elham M. A. Dannoun, Shujahadeen B. Aziz, Sozan N. Abdullah, Muaffaq M. Nofal, Khaled H. Mahmoud, Ary R. Murad, Ranjdar M. Abdullah, Mohd. F. Z. Kadir

**Affiliations:** 1 Associate Director of General Science Department, Woman Campus, Prince Sultan University, P.O. Box 66833, Riyadh 11586, Saudi Arabia; elhamdannoun1977@gmail.com; 2 Hameed Majid Advanced Polymeric Materials Research Lab., Physics Department, College of Science, University of Sulaimani, Qlyasan Street, Sulaimani 46001, Kurdistan Regional Government, Iraq; ranjdar.abdullah@univsul.edu.iq; 3 Department of Civil engineering, College of Engineering, Komar University of Science and Technology, Sulaimani 46001, Kurdistan Regional Government, Iraq; 4 Department of Chemistry, College of Science, University of Sulaimani, Qlyasan Street, Sulaimani 46001, Kurdistan Regional Government, Iraq; sozan.abdulla@univsul.edu.iq; 5 Department of Mathematics and General Sciences, Prince Sultan University, P.O. Box 66833, Riyadh 11586, Saudi Arabia; muaffaqnofal69@gmail.com; 6 Department of Physics, College of Khurma University College, Taif University, P.O. Box 11099, Taif 21944, Saudi Arabia; k.hussein@tu.edu.sa; 7 Department of Pharmaceutical Chemistry, College of Medical and Applied Sciences, Charmo University, Chamchamal, Sulaimani 46023, Iraq; ary.murad@charmouniversity.org; 8 Centre for Foundation Studies in Science, Physics Division, University of Malaya, Kuala Lumpur 50603, Malaysia; mfzkadir@um.edu.my

**Keywords:** solid polymer electrolyte, impedance, transport properties, FTIR study, electrochemical double-layer capacitor device

## Abstract

The solution cast process is used to set up chitosan: dextran-based plasticized solid polymer electrolyte with high specific capacitance (228.62 F/g) at the 1st cycle. Fourier-transform infrared spectroscopy (FTIR) pattern revealed the interaction between polymers and electrolyte components. At ambient temperature, the highest conductive plasticized system (CDLG–3) achieves a maximum conductivity of 4.16 × 10^−4^ S cm^−1^. Using both FTIR and electrical impedance spectroscopy (EIS) methods, the mobility, number density, and diffusion coefficient of ions are measured, and they are found to rise as the amount of glycerol increases. Ions are the primary charge carriers, according to transference number measurement (TNM). According to linear sweep voltammetry (LSV), the CDLG–3 system’s electrochemical stability window is 2.2 V. In the preparation of electrical double layer capacitor devices, the CDLG–3 system was used. There are no Faradaic peaks on the cyclic voltammetry (CV) curve, which is virtually rectangular. Beyond the 20th cycle, the power density, energy density, and specific capacitance values from the galvanostatic charge–discharge are practically constant at 480 W/Kg, 8 Wh/Kg, and 60 F g^−1^, for 180 cycles.

## 1. Introduction

In the creation of solid polymer blend electrolytes (SPBEs) for energy storage devices, both natural and synthetic polymer materials have been widely used as host polymers [1]. Metal salts can be dissolved in a polymeric matrix and then dissociated into metal cations and counter anions to make polymer electrolytes. These polymers are ionically conductive, and as a result, they have received a lot of interest because of their use in a variety of electrochemical devices [2].

Biopolymer materials (BPMs) demonstrated key features for use in electrochemical devices, high-energy-density batteries, sensors, and fuel cells, owing to some outstanding characteristics such as natural abundance; non-toxicity; renewability; cost effectiveness; biodegradability; eco–friendliness; harmlessness; and the fact it can be attained easily from natural resources such as cell walls, plants, and animals. [3,4,5,6,7].

The natural resources of biodegradable polymers such as cellulose, chitosan, and carrageenan have recently engaged the attention of several research activities since they are nontoxic and simple to use in electrolyte synthesis [8]. Chitosan (CS), for instance, is a deacetylated chitin derivative that, together with cellulose, is one of nature’s most plentiful biopolymers [9]. CS is mostly derived from shrimp waste, and it is widely employed as a natural polymer in a variety of applications [10]. Chitosan is a common sorbent with a high affinity for transition metal ions due to its concentration in polar groups (NH_2_ and OH) inside chains that act as conjunction sites [11]. The ability of chitosan to be molded into a variety of shapes is seen as a significant characteristic, with various shapes ranging from hydrogels to porous scaffolds to films [12].

Dextran is a natural polymer formed by the fermentation process of the bacteria *Leuconostocmesenteroides*, which has been widely studied in this field [13,14]. One technique to make a biodegradable polymers host with high ionic conductivity, excellent thermal characteristics, strong mechanical strength, and minimal toxicity is to combine two or more natural polymers [15]. In addition to polymer blending, plasticizers such as glycerol, polyethylene glycol (PEG), ethylene carbonate (EC), and poly (ethylene carbonate) (PEC) can improve conductivity. Glycerol has been shown to be compatible with the majority of natural polymers. Biopolymer and biodegradable polymer-based electrolytes have been broadly utilized in electrochemical devices in recent decades [16,17,18,19]. The ionic conductivity of polymer electrolyte can be increased when plasticizers are used [20]. Other studies of polymer electrolyte have shown that employing glycerol as a plasticizer works well for the system [4,21,22]. This is owing to glycerol’s ability to build more ionic channels inside the electrolyte, which has a significant impact on an electrochemical device’s performance [23]. For example, carbon nano–onions (CNO) and boron-doped diamond (BDD) have both been used as supercapacitor electrode materials [24,25].

These electrode materials, however, have a high manufacturing cost, which limits their applicability, as well as a smaller active surface area. Because of their high specific surface area, thermal stability, electronic conductivity, and electrochemical stability, activated carbon-based electrode materials are preferred in EDLCs. Activated carbon is simple to use and compatible with a wide range of solvents, binders, and electronic conductors [26,27,28].

The energy storage process for EDLC is a non-Faradaic routine in which there is no electron exchange and then ions form a double layer on carbon-based electrodes [27,29,30]. It was shown that EDLC has a reasonably higher power density; higher reversibility; and cheap, safer, and easier fabrication procedures than a Faradaic capacitor or pseudo–capacitor [31,32]. Andrade and coworkers increased the conductivity (4.7 × 10^−4^ S/cm) of a pectin–lithium perchlorate (LiClO_4_) electrolyte by adding glycerol [33].

Due to its promising features, a 60:40 combination of CS and dextran was chosen as the polymer host in this study [14,34]. Using numerous characterization techniques, this work aims to investigate the influence of varied glycerol concehntrations on the CS: dex: LiClO_4_ electrolyte system, with the highest conducting system being utilized in an electrochemical double-layer capacitor (EDLC) device.

## 2. Experimental

### 2.1. Materials and Sample Preparation

Dextran (Dex) and chitosan (CS) powders were purchased from Sigma−Aldrich (Kuala Lumpur, Malaysia) as the raw materials. The CS: Dex blended polymer is made by dissolving 40 weight percent Dexand 60 weight percent CS separately in 1 weight percent acetic acid (50 mL) for 1.5 h at room temperature. After that, both solutions were blended and agitated for 4 h to ensure that the blending solution was homogeneous. Forty wt.% LiClO_4_ was dissolved in the solution in a separate container. Then, in fourteen steps, glycerol in various concentrations ranging from 14 to 42 weight percent was added to the CS–Dex–LiClO_4_ blended electrolyte solution, which was constantly agitated to achieve plasticized CS-Dex-LiClO_4_ electrolytes. Polymer blend electrolytes were coded as CDLG–1, CDLG–2, and CDLG–3 for CS–Dex–LiClO_4_ containing 14, 28, and 42 wt. percent glycerol, respectively. Consequently, the electrolyte series was left at room temperature while the casting process in the labeled Petri dishes was carried out. The produced film was dried further by transferring it to a desiccator filled with silica gel for 12 days at ambient temperature with around 20% humidity. As a result of adopting this process, a series of solvent-free films were obtained.

### 2.2. Test Methods

#### 2.2.1. Electrical Impedance Spectroscopy (EIS)

The electrical characteristics of materials and their relationship with electronically conducting electrodes can be determined using complex impedance spectroscopy. Under spring pressure, solid polymer electrolyte (SPE) films with thickness 0.027 cm were sliced into compact discs (2 cm diameter and thickness 0.5 cm) and sandwiched between two stainless steel electrodes.

The impedance of the films was determined using the HIOKI 3531 Z Hi–tester (No. 1036555, Hioki, Nagano, Japan), which was connected to a computer, over a frequency range of 50 Hz to 5000 kHz. At room temperature, measurements were also taken.

The software calculates the real (*Z*_r_) and imaginary (*Z*_i_) parts of impedance and controls the measurements. The bulk resistance (*R*_b_) was calculated from the intersection of the plot with the real impedance axis, and the *Z*_r_ and *Z*_i_ data were shown as a Nyquist plot. The conductivity can be estimated using the equation below [35,36]:

(1) $$\sigma_{dc}= [\frac{1}{R_{b}}]\times [\frac{t}{A}]$$

**Scheme 1:**
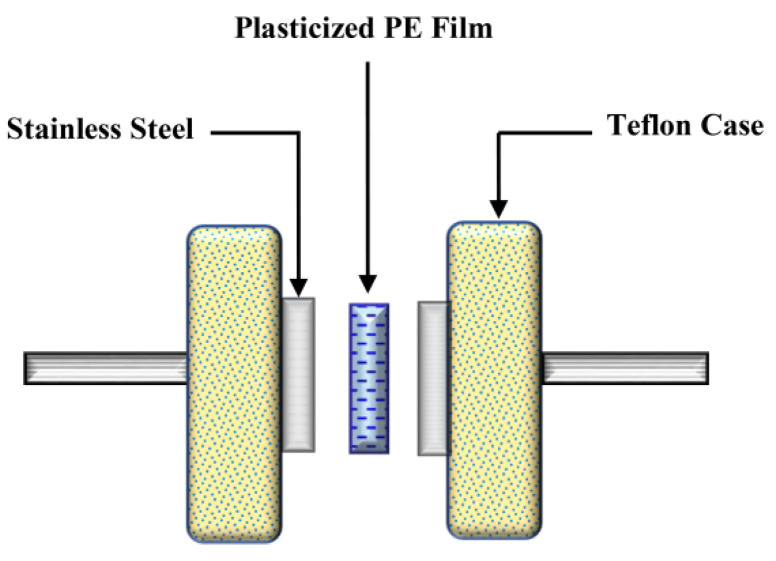
EIS measurement setup.

In Equation (1), *t* is the thickness of the film, *R*_b_ is the sample’s bulk resistance, and *A* is the active area. The average thickness of the plasticized systems is 0.026 cm. Schematically, the EIS measurement diagram is shown in Scheme 1.

#### 2.2.2. Fourier-Transform Infrared Spectroscopy (FTIR)

FTIR spectroscopy was used to inspect the interaction of numerous components of electrolytes, such as polymers, salt, and plasticizer. For this study, a Perkin Elmer Spotlight 400 spectrometer (Malvern Panalytical Ltd., Malvern, UK) with a resolution of 1 cm^−1^ (450 cm^−1^–4000 cm^−1^) was used.

## 3. Results and Discussion

### 3.1. Impedance Study

**Figure 1:**
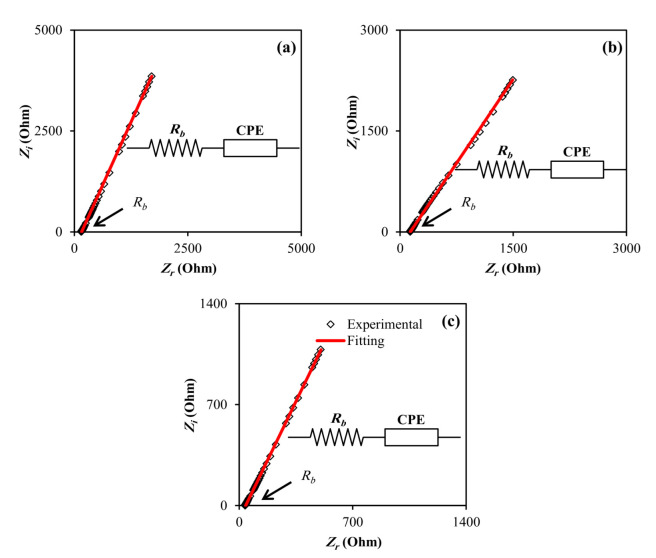
EIS plot for (**a**) CDLG–1, (**b**) CDLG–2, and (**c**) CDLG–3 plasticized electrolyte films.

It is worth noting that the charge transport process in composite materials is grave from both a theoretical and technical standpoint. Impedance scope is one of the most effective methods for distinguishing and comprehending charge transport in complicated materials [37]. Complex impedance plots (CIP) are useful parameters to investigate electrical properties of the polymeric materials since they help one to comprehend structure–property relationships [38]. The impedance plots for all of the samples are shown in Figure 1. The electrolyte/electrode interface might be regarded as a capacitance because blocking electrodes were utilized in the impedance study. When the capacitance is flawless, the impedance map should exhibit a vertical spike in the low-frequency zone.

Nonetheless, instead of the vertical spike, a spike inclined at an angle less than 90° was discovered, which might have been due to the irregularity of the electrolyte/electrode interface or double layer capacitances at blocking electrodes [39,40]. The bulk resistance was calculated by intersecting the spike with the impedance plot’s real axis. This is possible because when the phase angle is close to zero, the ionic conductance dominates the complex impedance [41]. Moreover, the spike pattern in the low-frequency band is a diffusion process characteristic [42]. Figure 1 shows that when the glycerol concentration increased, the bulk resistance (*R*_b_) decreased. The elimination of the semicircular component of complex impedance graphs in the high-frequency zone at high temperatures suggests that the conduction is primarily attributable to ions [43,44].

Using the *R*_b_ value and the sample size, Equation (1) was used to calculate the sample conductivity. The computed DC conductivity for all of the samples is shown in Table 1. Blend electrolytes have a high DC conductivity, which makes them ideal for EDLC. The representation of impedance plots via electrical equivalent circuits (EECs) can provide more information regarding the electrical properties of the blend electrolyte samples. It is feasible to predict the bulk resistance and circuit elements by modeling impedance plots.

The EECs model is universally used for fitting, or in the analysis of impedance spectroscopy, since it is effortless, rapid, and offers a whole observation of the system [45]. The recorded impedance graphs can be implicit in terms of the equivalent circuit for the charge carriers in the sample, which includes *R_b_*. Z_CPE_’s impedance may be written as [46,47]:

(2) $$Z_{CPE}= [\frac{\cos [\frac{\pi n}{2}]}{Y_{m}\omega^{n}}-j\frac{\sin [\frac{\pi n}{2}]}{Y_{m}\omega^{n}}]$$

where *Y_m_* denotes the CPE capacitance, *n* denotes the deviation of the vertical axis of the plot in complex impedance graphs, and ω denotes the angular frequency. Lastly, for the corresponding circuit (insets of Figure 1), the real (**Z*_r_*) and imaginary (**Z*_i_*) values of complex impedance (Z*) can be represented as:

(3) $$Z_{r}=R+\frac{\cos [\frac{\pi n}{2}]}{Y_{m}\omega^{n}}$$

(4) $$Z_{i}=\frac{\sin [\frac{\pi n}{2}]}{Y_{m}\omega^{n}}$$

The EEC fitting parameters are illustrated in Table 1. The semicircle fades in the Cole–Cole figure, meaning only the polymer’s resistive component is dominant [48].

Since the impedance data consist just of a spike, the *D* can be determined using the equation below [48]:

(5) $$D=D_{0}\exp\left\{-0.0297\;{ [\ln D_{0}]}^{2}-1.4348\ln D_{0}-14.504\right\}$$

where:

(6) $$D_{0}=\frac{4k^{2}l^{2}}{R_{b\;\;}^{4}\times \omega_{min}^{3}}$$

where *l* denotes sample thickness and *ω_min_* denotes the angular frequency that corresponds to *Z_i_*’s lowest value. Table 2 shows the electrolyte system’s ion transport parameter values. The Nernst–Einstein equation can be used to compute the ionic species’ mobility (*µ*) [49],

(7) $$\mu =\frac{e\times D}{K_{B}\times T}$$

where *T* and *K_b_* have normal meanings.

The conductivity ($\sigma_{DC}$) can alternatively be calculated using the following formula:

(8) $$\sigma_{DC}=n\times e\times \mu$$

As a result, the charge number density (*n*) can be calculated using the equation below [50]:

(9) $$n=\frac{\sigma_{DC}\times K_{b}\times T\times \tau_{2}}{{\left(e\times K_{2\;}\times \varepsilon_{0}\times \varepsilon_{r}\times A\right)}^{2}}$$

From Table 2, with the addition of glycerol, the *D* value increases considerably from 14 wt. percent to 48 wt. percent. It is also worth noting that the comparable tendency is visible for *μ* where the value rises. The upgrading in chain flexibility caused by glycerol can be attributed to the grow in *μ* and *D*; whilst the quantity of glycerol added is amplified, the *μ*, *D*, and *n* values rise, indicating that the conductivity rises. This is owing to the count of glycerol, which causes further salts to dissociate into free ions, increasing the number density of ions.

### 3.2. FTIR Study

**Figure 2:**
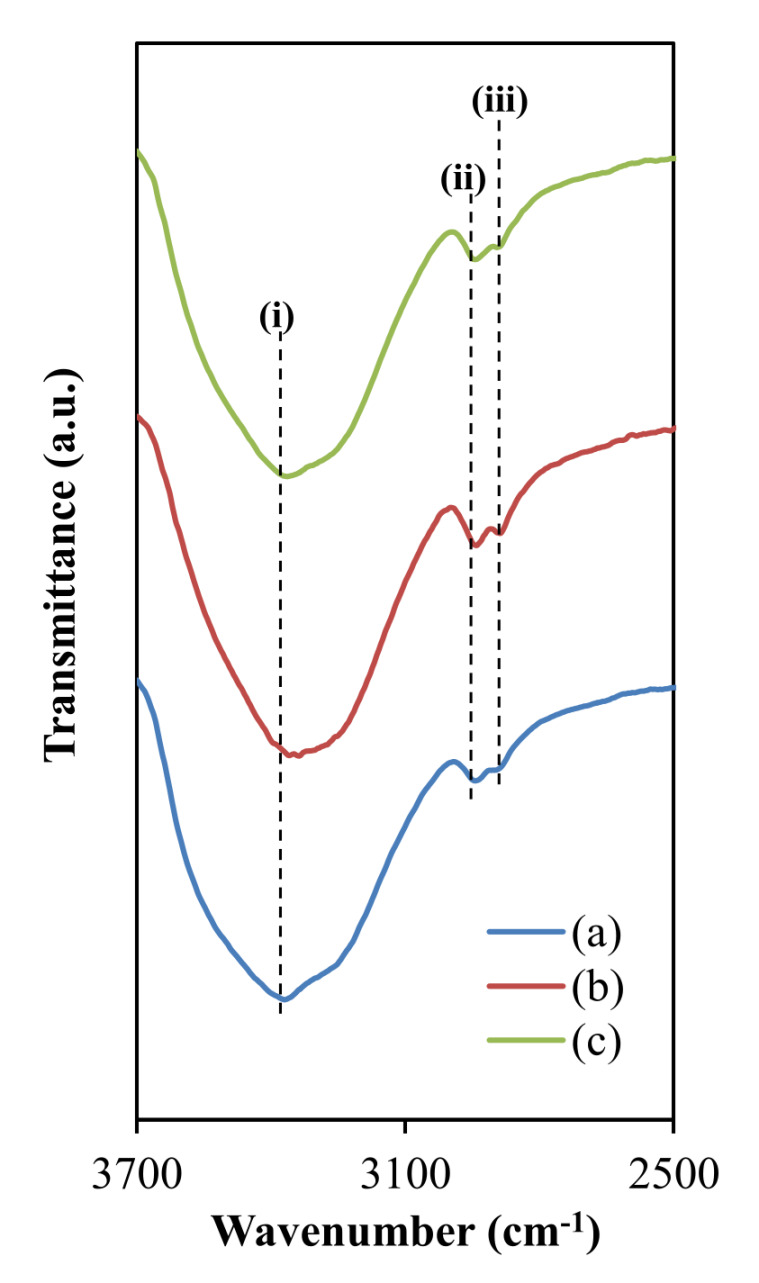
FTIR spectra at a wave number between 2500 and 3700 cm^−1^ for (**a**) CDLG–1, CDLG–2, and CDLG–3 plasticized electrolyte films.

As illustrated in Figure 2, the O–H stretching peak is positioned at (i) (3412 cm^−1^). The intensity of the O–H band peaks represents the complexation that occurs inside the electrolyte, which improves ionic dissociation and hence increases ionic conductivity [7,51]. Additionally, as shown in Figure 2, the C–H symmetrical stretching and C–H asymmetrical stretching are clearly visible at (ii) 2959 cm^−1^ and (iii) 2918 cm^−1^, respectively [52,53]. The intensity of these peaks improved as the concentration of glycerol increased, indicating the complex growth of glycerol in electrolytes [54].

**Figure 3:**
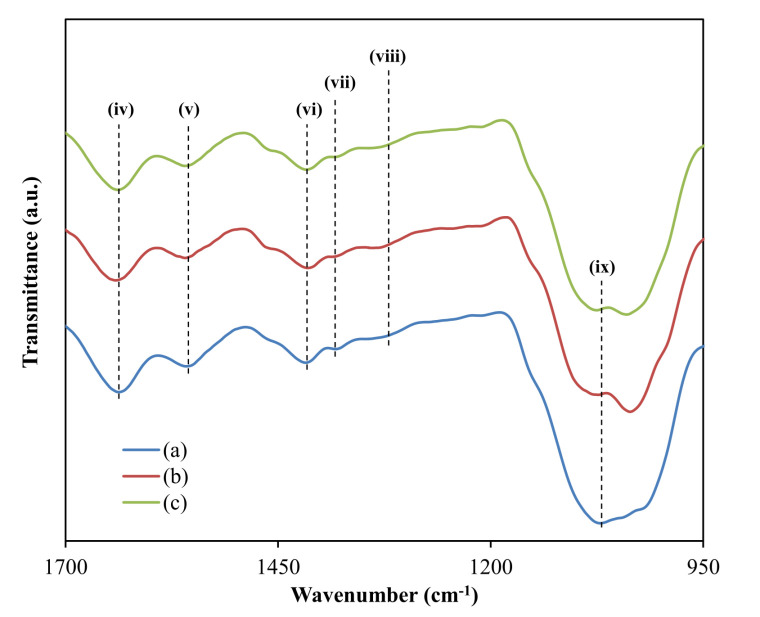
FTIR spectra at a wave number between 950 and 1700 for (**a**) CDLG–1, (**b**) CDLG–2, and (**c**) CDLG–3 plasticized electrolyte films.

Furthermore, as shown in Figure 3, the peaks at (iv) 1648 cm^−1^ and (v) 1564 cm^−1^ represent the electrolyte’s carboxamide and amine bands, respectively [55,56]. Moreover, the CH_2_ scissoring and C–H bindings are responsible for the peaks seen in Figure 3 at (vi) 1422 cm^−1^, (vii) 1390 cm^−1^, and (viii) 1327 cm^−1^, respectively [57,58,59,60]. Finally, the steep peak at (ix) 1077 cm^−1^ (Figure 3) could be attributable to C–O stretching, which has been observed by Mejenom and coworkers [61] and Poy and coworkers [62]. When the concentration of glycerol increases, the band peak widens, resulting in a tiny peak at 1044 cm^−1^, which also reflects the interaction inside the polymer matrix.

**Figure 4:**
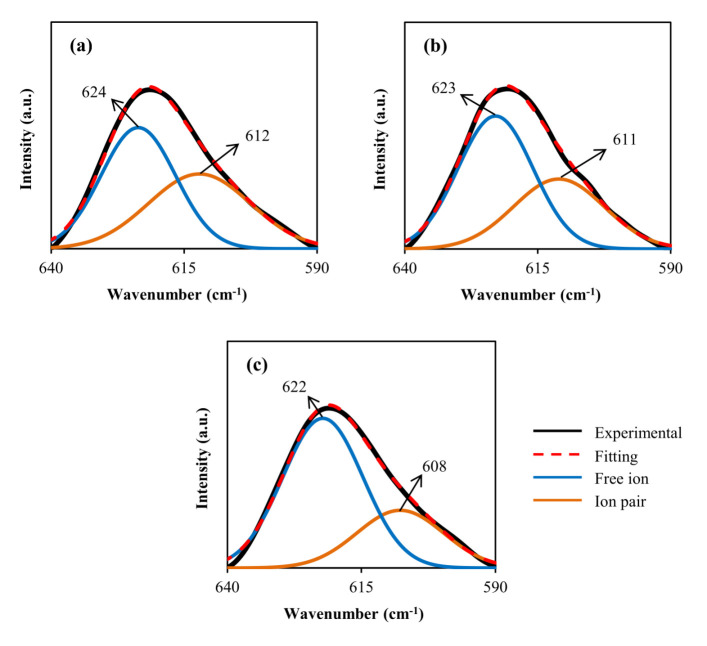
Deconvolution of FTIR spectra at a wave number between 590 and 640 cm^−1^ for (**a**) CDLG–1, (**b**) CDLG–2, and **(c)** CDLG–3 plasticized electrolyte films.

Figure 4 illustrates the deconvolution of FTIR spectra at a wave number between 590 and 640 for cm^−1^. The number density (*n*), ionic mobility (*µ*), and diffusion coefficient (*D*) based on the percentage of free ions were revealed using the FTIR method to more maintain the ionic conductivity research.

The Gaussian–Lorentzian function was used to extract overlapping peaks and adjust the curves‘ baselines using the deconvolution approach. The (ClO_4_) band’s FTIR peak at 650 to 600 cm^−1^ is frequently employed to inspect ion–ion interactions in polymer electrolytes containing LiClO_4_ salt [63,64,65,66]. The peak of the (ClO_4_) band divided into two peaks at 624 cm^−1^ and 635 cm^−1^ shows that mainly two unlike types of ClO_4_ anions survive in this complex, which is well established [63,64,65,66].

Salomon et al. proposed that the existence of Li^+1^ is connected with the (ClO_4_) band centered between 630 and 635 cm^−1^. ClO_4_^−1^ contact–ion pairs are responsible for the band centered at about 623 cm^−1^, whereas free/unpaired ClO_4_ anions are responsible for the band centered at about 623 cm^−1^ [64,66]. The peak characteristic for ‘‘free” ClO_4_ is substantially greater than that of contact-ion pairs, as seen in Figure 4. This is due to the glycerol plasticizer’s ability to aid in the dissolution of LiClO_4_ salt through the CS: Dex mix matrix. The following formulae can be used to calculate the percentage of free ions and contact ion pairs [30,67,68,69]. Table 3 displays the percentages of ion carriers.

(10) $$\mathrm{Percentage}\;\mathrm{of}\;\mathrm{free}\;\mathrm{ion}\;\left(\%\right)=\frac{A_{f}}{A_{f}+A_{c}}\times 100\%$$

(11) $$\mathrm{Percentage}\;\mathrm{of}\;\mathrm{contact}\;\mathrm{ion}\;\mathrm{pairs}\;\left(\%\right)=\frac{A_{c}}{A_{f}+A_{c}+A_{a}}\times 100\%$$

where *A_f,_* and *A_c,_* are the area of the peaks of the free ions and the contact ion pair, respectively.

The charge carrier parameters can be calculated using the percentage of free ions of each electrolyte and the relationship illustrated below (12)–(14) [30,67,68,69].

(12) $$n=\frac{M\times N_{A}}{V_{Total}}\times \left(free\;ion\;\%\right)$$

(13) $$\mu =\frac{\sigma }{n\;e}$$

(14) $$D=\frac{\mu kT}{e}$$

where *M* is the number of glycerol moles, *N_A_* denotes Avogadro’s number, and *V*_Total_ denotes the whole volume of the polymer electrolytes. The Boltzmann constant and elementary charges are denoted by *k* and *e*, respectively, while *T* denotes the temperature in Kelvin. Table 4 illustrates the values of transport parameters. It can be shown that raising the glycerol concentration in the polymer electrolyte increases the number density of ions and ionic mobility significantly, as shown in Table 4. It also induces an increase in the diffusion coefficient of ions. Table 2 shows that the ion transport parameters calculated using impedance theory coincide with those calculated using FTIR theory in Table 4. It can be concluded that, as the number density, diffusion coefficient, and mobility increased, the glycerol content increased.

### 3.3. Electrochemical Properties

#### 3.3.1. TNM Study

Electrochemical devices that employ ions as current carriers rely on solid polymer electrolytes [70]. The progress of safe electrolytes, mainly solid polymer electrolytes (SPEs), is an imperative component of electrochemical device research. Despite the fact that this compound system has been around since the late 1970s, it is still a hot topic [71,72].

**Figure 5:**
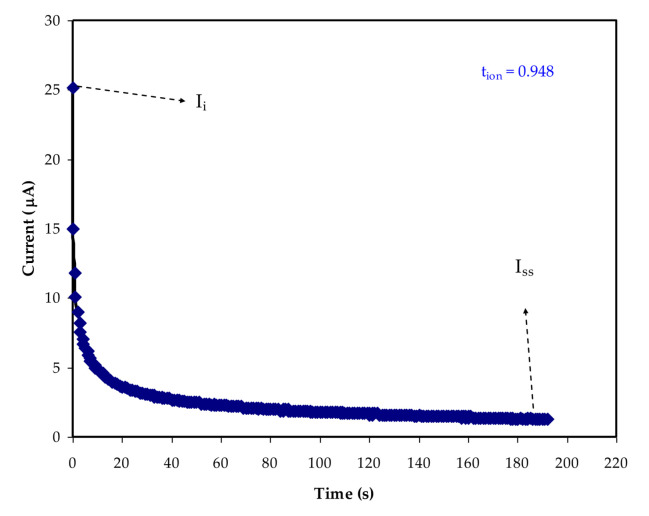
Polarization curve of current for the highest conducting sample.

It is essential to understand the transference number analysis (TNM) and linear sweep voltammetry (LSV) to employ the polymer electrolyte for application. The main charge carrier species in a polymer electrolyte must be determined, which can be done via TNM. The ionic transference number of the sample was determined using the DC polarization technique (*t_ion_*). The method includes applying DC voltage to the sample below its decomposition potential, then examining the ensuing currents in proportion to time as shown in Figure 5 [73].

To make the measurement easier, the polymer electrolyte with the highest conductivity was positioned between a couple of stainless-steel blocking electrodes (SS), and the ion (*t_ion_*) and electron (*t_el_*) transference numbers were calculated using the equation below [74,75]:

(15) $$t_{ion}=\frac{I_{i}-I_{ss}}{I_{i}}$$

(16) $$t_{ion}=1-t_{el}$$

where the steady-state current is I_ss_ and the initial current is I_i_. At 25 µA, the I_i_ can be seen. The participation of both ions and electrons at the beginning stage accounts for the considerable value of initial current. Because the electrodes are stainless steel, which is known to impede ions, the current drops dramatically before becoming constant at 2 μA [76].

The behavior of an ionic conductor is represented by this phenomenon [77]. The results show that ions are the major charge carriers within the polymer electrolyte, with *t_ion_* = 0.948 and *t_el_* = 0.052. The closeness of *t_ion_* to 1, the ideal value, is a particularly interesting result, indicating that the transport mechanism of the produced electrolyte film is largely ionic [73].

This is due to the potential of Li^+^ cations dissociating from the coordinating sites of CS: Dex polymer chains, and charge transport in the polymer blend is thus mostly due to cationic motion [78]. If the *t_ion_* value is close to unity, the ions contribute to the system’s carrying charge, according to Shukur et al. [79]. Mohan et al. [80] and Tang et al. [81] reported similar observations.

#### 3.3.2. LSV Study

**Figure 6:**
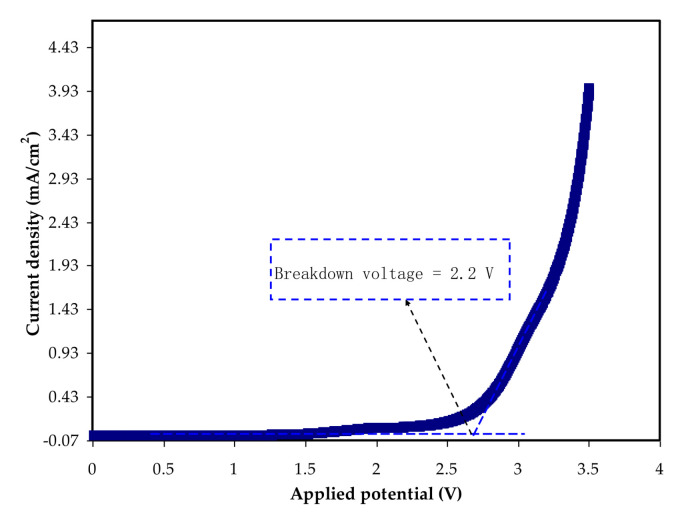
LSV plot for the highest conducting system.

The LSV technique is used to determine an electrolyte’s breakdown voltage at ambient temperature [82]. The potential casement is a vital aspect and serious parameter in determining the requirement of some electrochemical devices, such as capacitors and batteries [50]. Figure 6 displays the LSV plot for the CDLG–3 electrolyte up to 4.0 V. Figure 6 shows a plot of current density against voltage that shows an increase in voltage lacking any current flows until 2.2 V, indicating that the electrochemical reaction in the electrolyte did not happen at this point [52].

This event implies that the electrolyte’s breakdown voltage is 2.2 V, which is similar to what has been described in the literature [19]. As a result, this demonstrates the electrolyte’s potential to give a promising performance in an EDLC that is generally run at 1.0 V [29,83].

#### 3.3.3. CV Analysis

For the energy storage process, typical EDLCs use a non-Faradaic mechanism that involves non-redox processes. This condition was investigated using cyclic voltammetry (CV) analysis at various sweep rates (*a*) to examine how it affects the specific capacitance (*C_cyc_*) calculated using the equation below [4,84]:

(17) $$C_{cyc}=\int_{V_{i}}^{V_{f}}\frac{I\left(V\right)dV}{2ma\;\left(V_{f}-V_{i}\right)}$$

**Figure 7:**
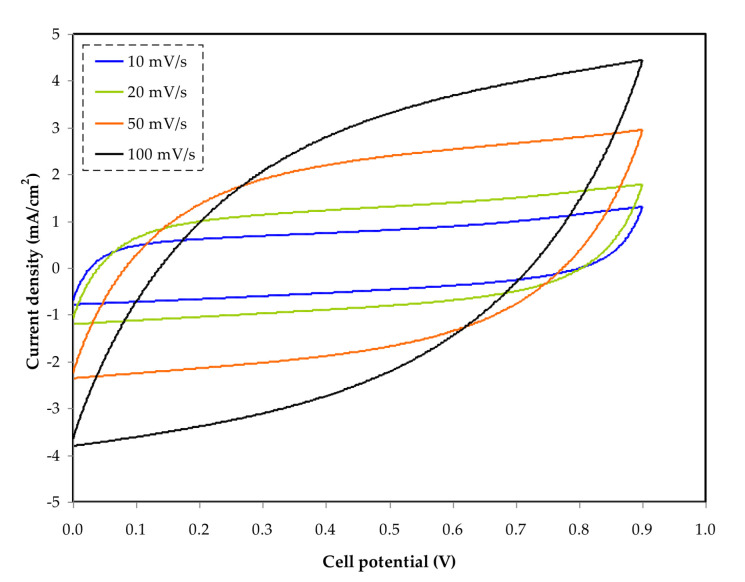
Cyclic voltammetry (CV) at various scan rates of 10, 20, 50, and 100 mV s^−1^.

The area of the CV plot in Figure 7 utilizes the integration function via Origin 9.0 software. The values of *V_f_* and *V_i_* are 0.9 V and 0 V, respectively. Table 5 shows the *C_cyc_* values measured at 10, 20, 50, and 100 mV/s. The specific capacitance is clearly influenced by sweep rates, as evidenced by these data.

The flow of cations and anions in the electrolyte is unstable at high sweep rates, such as 100 and 50 mV/s, causing incorrect charge double-layer formation at the surface of AC electrodes. This condition also results in current–dependent potential, which explains the form of the leaf at high sweep rates [85].

As shown in Figure 7, the CV curves show how the shape of the curve changes from rectangular to leaf-like as the scan rate increases. The presence of porosity in the carbon electrodes, as well as the internal resistance present throughout the measurement, creates these alterations [86].

The correct polarization procedure can be carried out at a slower sweep rate. This situation causes current to be independent of potential, resulting in a rectangular CV plot [87]. The curves did not show any noticeable peaks, indicating that the system did not submit to any reduction or oxidation reactions.

During the charging process, the anions in the system pour towards the positive electrode; the positive electrode resists the cations, causing the cations to draw to the negative electrode. During this process, the electric field snatches the electrode and electrolyte to hold the electrons and ions, respectively [88].

The development of double-layer charge to store potential energy on the carbon electrode surfaces is explained by this entire process [89].

#### 3.3.4. Galvanostatic Charge–Discharge Properties

**Figure 8:**
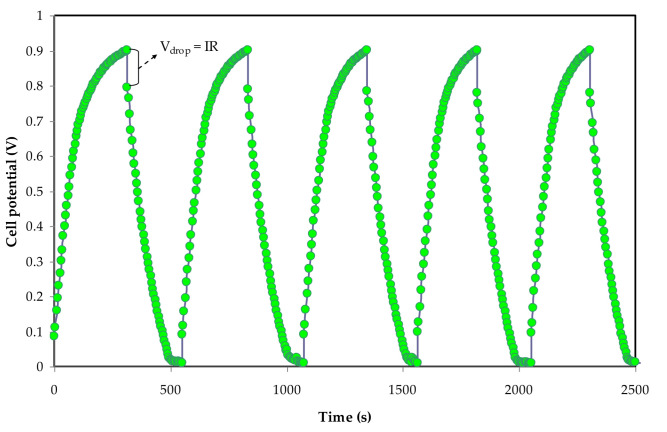
Galvanostatic charge–discharge curve for the constructed EDLC.

The cyclic discharge preserve can be used to assess the electrochemical capacitance of a substance. Figure 8 illustrates the galvanostatic charge–discharge curves of the built EDLC cells at ambient temperature for CS: Dex doped with 40 wt. percent LiClO_4_ and glycerolized with 42 wt. percent. The EDLC cells also demonstrate linear discharge manners with a tiny ohmic loss, meaning a non-Faradic capacitive charge-storage mechanism [90]. The *C_spe_* of EDLC cells are able to be determined using the subsequent equation [48,49].

(18) $$C_{spe}=\frac{i}{xm}$$

**Figure 9:**
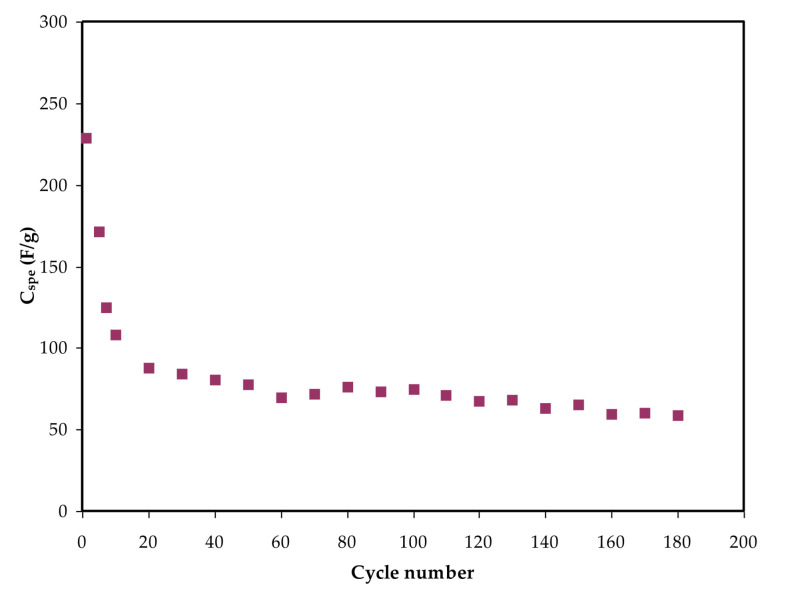
Specific capacitance for the constructed EDLC throughout 180 cycles.

The applied current is shown here as *i*. The slope of the discharge curve is (x). The *C_spe_* is plotted against the cycle number in Figure 9. The capacitance charge achieved in this system is moderately superior to that found in earlier studies. The first cycle’s calculated *C_spe_* value is 235 F/g, and the second cycle’s *C_spe_* value is roughly 60 F/g.

When judged against the capacitance values of 2.6–3.0 and 1.7–2.1 F/g that were obtained for EDLC cells using Mg- and Li-based PEO polymer electrolytes integrated with ionic liquids [85], the capacitance generated in this work is of considerable attention. Beyond the 20th cycle, the capacitance is nearly constant across 180 cycles.

Other studies have found a significant drop in *C_spe_* over a large number of cycles. The researchers theorized that the loss of these electrochemical properties could be due to the creation of ion aggregates. When it comes to mobile ions, they tend to be aggregated or paired together after fast charge and discharge operations.

Considerably, the ion pairs have the potential to inhibit the polymer electrolyte’s ionic migration, affecting the rate of ions adsorption via the carbon pores. As a result, ion adsorption generation at the electrode–electrolyte interface is reduced. As a result, the energy and power density of EDLC cells, in addition to their specific capacitance, are inversely proportional to the cycle number [91]. Further researchers reported, for instance, 4 F/g, 4.3 F/g, 8.4 F/g, and 6.5–15 F/g for PEO, PVDF–HFD, polyurethane, and PVA–cellulose-based electrolytes inserted with lithium salts, respectively [83]. As a result, *C_spe_* is higher than previous results for polymer-based electrolytes incorporating Li^+^ ion salts published in the literature.

**Figure 10:**
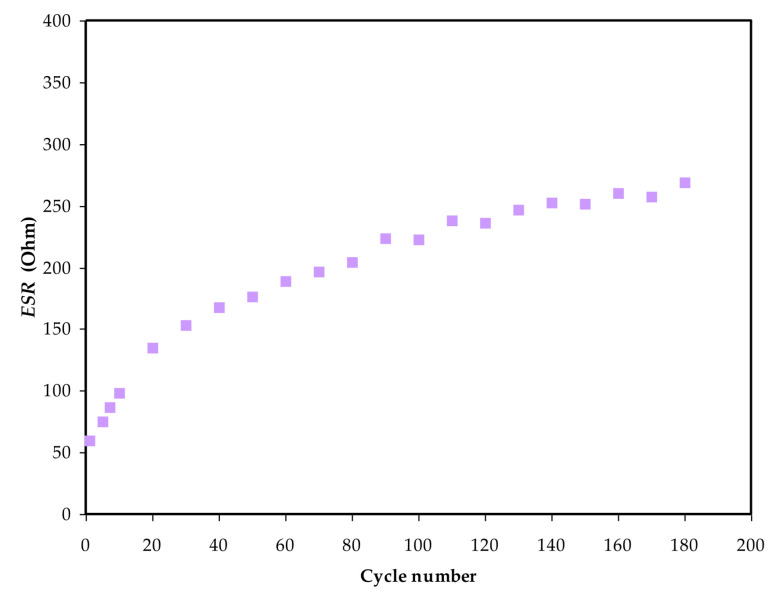
The ESR values versus cycle number of the EDLC assembly.

As shown in Figure 10, a little voltage drop, *V_d_*, is also seen in the charge–discharge graph. The internal resistance within the system during the charge–discharge operations, also known as equivalent series resistance, ESR, is responsible for the creation of *V_d_*. The applied current, *i* of 1 mA, will be used to determine this parameter, which can be represented as [48,50]:

(19) $$ESR\;=\frac{V_{drop}}{i}$$

Figure 10 shows the obtained ESR values for 180 cycles. The system is seen to retain an ESR in the choice of 55 to 272 Ω throughout the entire operation. This outcome indicates that the EDLC had a little consistent internal resistance for 180 cycles, simplifying the electrostatic process between the ions and charged electrode [92].

The constructed EDLC’s energy density (*E_d_*) can be estimated using the equation below [48,74]:

(20) $$Ed=\frac{C_{2}\times V}{2}$$

**Figure 11:**
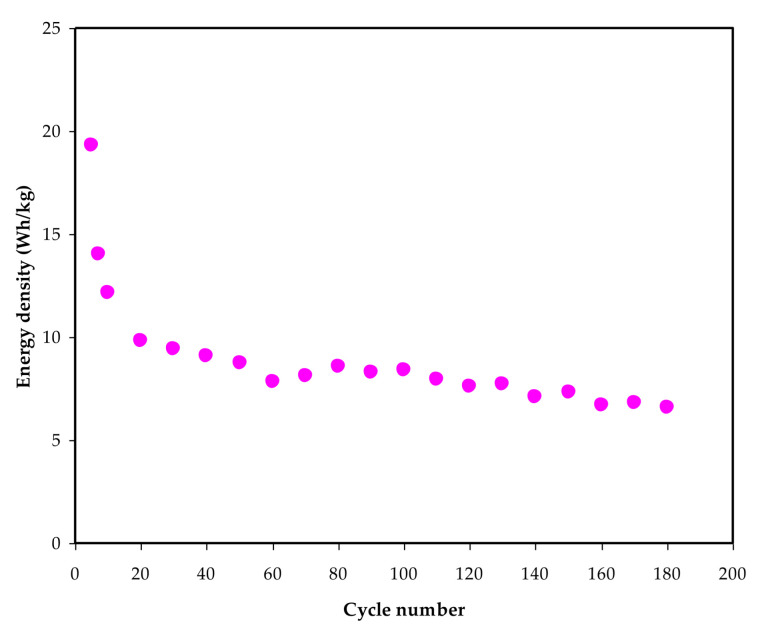
Energy density (*E_d_*) versus cycle number for 180 cycles.

V Equals 1 V. *E_d_* is 18.4 Wh/kg on the first cycle, as shown in Figure 11. From the 60th to the 180th cycle, the *E_d_* drops to 7.5 Wh/kg before stabilizing at 6.2 Wh/kg on average. As a result, it may be inferred that after the 60th cycle, the energy barrier for ion conduction is nearly the same [93]. This work’s *E_d_* is similar to other EDLCs that have been reported utilizing numerous materials.

According to Hina et al. [94], the value of *E_d_* for their EDLC varies between 9.82 and 21.6 Wh/kg and relies on the content of lithium triflate (LiTf). Mazuki and coworkers used EDLC to obtain 1.19 Wh/kg for the CMC-PVA-NH_4_Br combination [95]. The existence of plasticizer in this work could explain the high *E_d_*. Biopolymer-based electrolytes are crucial for energy storage applications, according to the findings of this study.

**Figure 12:**
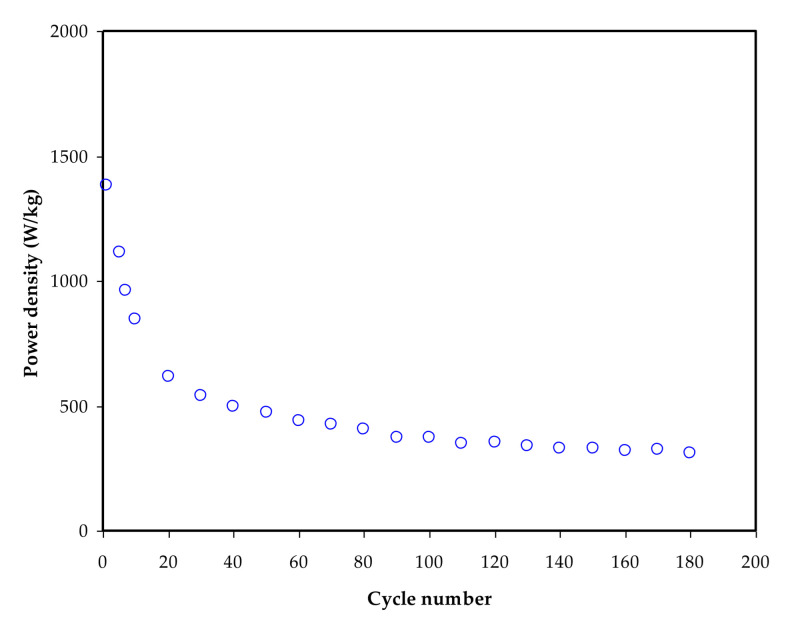
Power density (*P_d_*) versus cycle number for 180 cycles.

Furthermore, if ESR values are available, the power density (*P_d_*) values are determined, as shown in Figure 12. The power density (*P_d_*) of the constructed EDLC is depicted in Figure 12 and is computed using the equation below [4,48]:

(21) $$P_{d}=\frac{V^{2}}{4\times m\times R_{esr}}$$

The *P_d_* value is 1450 W/kg on the first cycle and lowers to 480 W/kg after the EDLC has completed 180 cycles. The *P_d_* pattern is in fine arrangement with the ESR plot’s trend. This is owed to electrolyte deterioration, which occurs when resistance rises, generating ionic recombination as a result of the swift charging and discharging mechanism, resulting in lower *P_d_* at a high cycle number [96]. The mass loading of active material in the production of EDLC has a clear impact on both *E_d_* and *P_d_* values. The improved electrochemical performance is said to be due to the low mass loading and comparatively low current [97].

## 4. Conclusions

The solution casting approach was used to prepare LiClO_4_: glycerol–based solid polymer electrolytes. The interaction of the electrolyte’s elements was determined using FTIR. The addition of 42 wt % glycerol improved the conductivity to 4.16 × 10^−4^. Both impedance and FTIR methods revealed that as glycerol levels increased, the number density (*n*), mobility (*μ*), and diffusion coefficient (*D*) of ions enhanced. TNM revealed that cations and anions made up the majority of charge carriers, with t_ion_ and t_e_ values of 0.948 and 0.052, respectively, for the CDLG–3.LSV revealed that the CDLG–3′s electrochemical stability potential was 2.2 V, indicating that the SPE could be used in an EDLC application. The CV curve has a rectangular form with no redox peaks, confirming the capacitive behavior of the ELDC. Beyond the 20th cycle, the power density, energy density, and specific capacitance values from the galvanostatic charge–discharge are practically constant at 480 W/Kg, 8 Wh/Kg, and 60 F g^−1^, respectively, for 180 cycles.

## Figures and Tables

**Table 1:** Ionic conductivity and the parameters fitting of the EEC.

Electrolyte	*p* (rad)	*R_b_* (Ω)	CPE (F)	*σ* (S cm^−1^)
**CDLG-1**	0.37	151	3.53 × 10^−6^	1.02 × 10^−4^
**CDLG-2**	0.51	134	9.80 × 10^−6^	1.15 × 10^−4^
**CDLG-3**	0.39	37	1.36 × 10^−5^	4.16 × 10^−4^

**Table 2:** Ion transport parameter from impedance theory.

Electrolyte	*n* (cm^−3^)	*μ* (cm^2^V^−1^s^−1^)	*D* (cm^2^s^−1^)
**CDLG-1**	6.63 × 10^20^	9.62 × 10^−7^	2.47 × 10^−8^
**CDLG-2**	6.72 × 10^20^	1.07 × 10^−6^	2.74 × 10^−8^
**CDLG-3**	1.15 × 10^21^	2.26 × 10^−6^	5.80 × 10^−8^

**Table 3:** The percentages of ion species using FTIR method.

Electrolyte	Ions Pair (%)	Free Ions (%)
**CDLG-1**	45.8	54.2
**CDLG-2**	38.8	61.2
**CDLG-3**	29.4	70.6

**Table 4:** Ion transport parameters using FTIR theory.

Electrolyte.	*n* (cm^−3^)	*μ* (cm^2^V^−1^s^−1^)	*D* (cm^2^s^−1^)
**CDLG-1**	2.11 × 10^22^	3.02 × 10^−8^	7.89 × 10^−10^
**CDLG-2**	2.25 × 10^22^	3.19 × 10^−8^	8.33 × 10^−10^
**CDLG-3**	4.44 × 10^22^	5.84 × 10^−8^	1.52× 10^−9^

**Table 5:** Specific capacitance (F g^−1^) value at various scan rates.

Scan Rate	Capacitance (F/g)
100	26.06
50	42.34
20	64.01
10	75.90
